# Reduction of Radiation Risk to Interventional Cardiologists and Patients during Angiography and Coronary Angioplasty

**Published:** 2017-07

**Authors:** Mohsen Mohammadi, Leili Danaee, Effat Alizadeh

**Affiliations:** 1 *Department of Medical Radiation Science, School of Paramedicine, Shahid Beheshti University of Medical Sciences, Tehran, Iran.*; 2 *Department of Medical Physics, Faculty of Medicine, Tabriz University of Medical Sciences, Tabriz, Iran. *; 3 *Department of Medical Biotechnology, Faculty of Advanced Medical Sciences, Tabriz University of Medical Sciences, Tabriz, Iran.*

**Keywords:** *Radiation exposure*, *Cardiologists*, *Patients*

## Abstract

Radiation risk allied to invasive cardiology is relatively high, and protecting both patients and cardiologists is necessary. The aim of this review is to discuss how to better protect patients and cardiologists against radiation exposure. We performed a global search on PubMed, Science Direct, and Scopus databases via keywords of “interventional cardiologist”, “patient”, “radiation”, and “exposure” and then performed an overview of the main strategies for risk reduction among interventional cardiologists and exposed patients. The 1st line for protection is awareness of both radiation risk factors and exposure doses and how to manage and minimize exposure levels. In conclusion, radiation-attenuating techniques can effectively reduce occupational/treatment radiation exposure to both operators and patients in cardiology interventions.

## Introduction

Ionizing radiation is necessary for invasive cardiology procedures such as coronary angiography, percutaneous coronary intervention (PCI), and electrophysiological diagnostics or therapeutics.^1^ Radiological examinations are necessary for therapeutic processes.^2^^, ^^3^ About 12% of all radiological examinations are interventional cardiac procedures, leading to exposure to the highest radiation dose (up to 50% of the total collective effective dose). Therefore, such examinations are a serious cause for concern for interventional cardiologists and patients given the high exposure.^4^

According to the United Nations Scientiﬁc Committee on the Effects of Atomic Radiation (UNSCEAR) report in 2008, fluoroscopic procedures are considered the largest source of medical occupational exposure. The American Heart Association (AHA) Science Advisory specified the reference doses of cardiology examinations in 2009, and in 2010 the American College of Cardiology (ACC) committee also highlighted the need for the development of safer radiation techniques in cardiology.^5^

The mean radiation exposure for left ventriculography and coronary angiography is equivalent to about 300 chest X-rays, a peripheral artery intervention to 1500 to 2500, a coronary stent to 1000, and finally in a cardiac radiofrequency ablation from 9000 to several thousands. The cardiologist is the only person involved in most of the procedures, without any training regarding radiation protection.^6^ Furthermore, interventional cardiologists are usually exposed to the highest dose of ionizing radiation among the medical staff. It is estimated that the exposure per head per year of this group is 2 to 3 times higher than that of radiologists. Recent reports have documented that an experienced interventional cardiologist in high-volume centers has an exposure near to 5 mSv per year, which is not negligible from the carcinogenic point of view.^7^

The risk model for low-level exposures is based on a linear relationship between the dose of radiation and the long-term risk of carcinogenesis. The assessment of health risk in low doses has been controversial and is estimated from the extrapolation of the high-dose-derived dose-effect curve.^8^

In particular, in spite of the deepening scientific knowledge about the adverse health effects of radiation, there is a particular need to introduce new epidemiological approaches in radioprotection programs aiming at the health surveillance of the hospital staff exposed to ionizing radiations chronically.^9^

The cardiac catheterization laboratory is deemed a source of radiation. Radiation risks are divided into 2 types: the deterministic type, which causes effects after passing from a certain threshold (e.g., skin burns), and the stochastic type, which is a risk proportional to the dose of radiation received by the patient (e.g., malignancy and teratogenicity). Since procedures are becoming more complex, radiation exposure attenuation in the cardiac catheterization laboratory is vital. However, utilizing decreased doses results in lower image quality because of a diminished signal-to-noise ratio.^1^

Recently, Engin et al.^10^ (2005) studied the genomic instability of γ- and X-ray-exposed hospital personnel and showed that chronic exposure to ionizing radiation, even in lower levels than the accepted limit, could induce oxidative stress and increased apoptosis in comparison with nonexposed personnel.^11^

Scientific evidence gathered from both human and animal experiments has revealed that the risk associated with lower radiation doses is due to the stochastic effect. Thus, other factors such as low-dose hypersensitivity can intensify the consequences of the radiation, but adaptive responses against targeted and non-targeted damage may relieve the chronic damage.^12^^, ^^13^

According to previous studies, there are 2 ranges of the radiation dose for the staff in the coronary angiography department: 0.5 to 6 mSv (median effective dose = 1.3 mSv) and 1 to 11 mSv (median effective dose = 5 mSv).

The International Commission for Radiation Protection proposed a radiation dose that is “As Low As Reasonably Achievable” (ALARA) to keep radiation safe.^14^ The aim of this paper is to discuss strategies vis-à-vis radiation attenuation for patients and interventional cardiologists.

## Methods

We performed a literature review in PubMed, ScienceDirect, and Scopus databases using the terms “radiation”, “exposure”, “interventional cardiologist”, and “patient” as keywords. Additionally, we drew upon Boolean operators to connect and define the relationship between our selected search terms. We excluded studies before 1990 and reviewed strategies aimed at reducing radiation risks to interventional cardiologists and patients.

***Measuring occupational exposure at the cardiac laboratory***

Several quantities are used for characterizing the amount of radiation. The 1st quantity is called “air kerma”, defined as kinetic energy per mass unit. Its quantification is based on the energy that is transferred from non-directly ionizing particles for instance photons to charged particles. Gray (Gy) is the unit used for expressing air kerma: 1 Gy is equal to the energy transfer of 1 J/kg of the air.^15^ The other common quantities are fluoroscopy time, dose-area product (DAP), number of cine frames, effective dose, cine time, skin dose, and coronary dose. The coronary dose is defined as the dose to a coronary artery, and it can be measured using a dosimeter (catheter-based) during irradiation. The non-dosimetric quantity fluoroscopy time (expressed in min) is common for the evaluation of the patient’s dosimetry because it is the only readily available routine dose metric quantity in many interventional laboratories.^16^ However, the fluoroscopy time does not show any information on the skin entrance ports as well as the dose rate. The DAP, which is measured in Gy·cm^2^, is based on the dose in air for a certain plane by the area of the irradiating beam. It is not dependent on the distance from the X-ray source because the reduction of the dose with distance is equivalent to the increase in the area. It is the initial quantity for estimating the patient’s skin dose as well as establishing the stochastic risk to the patient by the effective dose.^17^^, ^^18^

International organizations have recommended the quantities and units that should be considered for occupational dosimetry. Also, national regulations have recommended specific requirements for staff dosimetry in interventional practices.^19^

Limitation of the dose for workers is the equivalent dose in a tissue (or organ) for an exposed part of the body and for whole-body exposure is the effective dose. The SI unit for the equivalent dose and the effective dose is the sievert (Sv).^20^ The maximum effective dose limit per year is 20 mSv, averaged over certain periods of 5 years. Nonetheless, it may not exceed 50 mSv in any 1 year. The European Union may necessitate stricter limitations. In Germany, a 400-mSv dose limit has been established for lifetime. Also, the individual state governments in the United States have established occupational dose limits, but the recommendations mostly developed by the National Council on Radiation Protection and Measurements (NCRP) are common.^16^^, ^^19^^, ^^20^

A 20% relative decrease in the operator dose for interventional cardiologists with low-rate fluoroscopy through PCI and directional coronary atherectomy is high; it could, therefore, save near to 6 years of radiation exposure over a 30-year occupation. The estimated lifetime extra risk for high-volume operators (occupational exposure of 5 mSv for 1 year) for either fatal or nonfatal cancers is near to 1%. 6 A 28% to 40% decrease in the operator radiation exposure could significantly reduce the risk. For trainees, the estimated radiation in the 1st year of training is 60% higher than that in the 2nd year. The reason for that higher exposure is the longer fluoroscopy time to position the catheter.^7^^, ^^21^^, ^^22^

***Reducing patient exposure to radiation***

Using decreased patient doses could result in a proportional reduction in the scatter dose to both the operator and other personnel in the interventional space. Based on phantom measurements and Monte Carlo simulations, a powerful correlation between the DAP and the effective dose has been found, providing evidence that using a simple conversion factor for effective dose estimation from DAP values is true.^15^^, ^^22^ The previously reported conversion factors are 0.185 mSv/ Gy·cm^2^, 0.183 mSv/ Gy·cm^2^, and 0.221 mSv/ Gy·cm^2^.^22^^, ^^23^

The ALARA optimization principles necessitate the minimization of the dose to balance the radiation risk by the interventional procedure benefit to the patient. Accordingly, it is essential that efforts be made to properly manage the radiation risk to the patient.^24^ The best approach for patient dose reduction is to lessen the beam-on time for both fluoroscopy and acquisition to the smallest possible amount. The most important procedure-related factors for governing the amount of scattered radiation are the orientation and movement of the beam.^25^^-^^27^

Fluoroscopy has a significantly higher proportion of whole air kerma during PCI than acquisition. The fluoroscopic air kerma rate shows more sensitivity to changes in angulation than the acquisition air kerma rate.^24^

***Modifying fluoroscopic views for operator radiation exposure reduction***

The amount of the radiation dose to both patients and operators is affected by tube angulation. Fluoroscopy shows more sensitivity to changes in angulation than acquisition. A reduction in patient and staff exposure to radiation requires the minimization of extreme angulations, whenever possible. In addition to beam angulation, another strong predictor of the total air kerma rate on the complex spatial map is the body surface area, which is developed by modeling based on multivariable regression.^15^^, ^^20^^, ^^28^^, ^^29^

Angulations of steep left anterior oblique (LAO) tubes are the most radiation intensive for both the operator and the patient. When the former LAO 60^°^/0^°^ view was replaced with the caudal right anterior oblique (RAO) 10^°^/30^°^, the fluoroscopic operator dose was reduced by around 75%. ^29^ LAO 060^°^ having cranial or caudal angulation 020^°^ is not recommended, and suggestions regarding how to replace common tube angulations by alternative safer angulations without significant loss of capture information are important. For any target structure, RAO or posterior anterior (PA) angulation with the appropriate amount can be used as an alternative to the standard LAO angulation.^15^ The LAO cranial projection, when used by a physician in the femoral approach for a patient, will result in the highest operator dose rate for the radiation scatter.^30^^, ^^31^ When cardiologists became aware of the relatively high scatter dose to operators after the LAO projection, the researchers observed a significant decrease in the use of the LAO projection. As a result, the average physician dose was decreased by about twofold.^32^ Raising awareness as regards radiation dose levels and radiation protection through educational programs is necessary.^28^^, ^^33^ It is recommended that the physician avoid being on the side of the tube during oblique and lateral projections because just by standing on the side of the image intensifier, the dose rate can be reduced by up to fivefold.^34^

A large reduction in the radiation exposure of the patient and, to a lesser degree, of the invasive cardiologist, along with an improvement in image quality, can be achieved by holding the image intensifier closer to the patient and the tube further away.^35^

***Pulsed fluoroscopy***

The reduction of the dose by factors 2 and 4 can be attained through maximum dose reduction, which utilizes X-ray techniques for 15 fps and 7.5 fps when it is in the case of 30 fps. The application of this strategy directs a reduction of the quality of the perceived image in lower frame rates in mode than the 30 fps mode due to the characteristics of human vision.^36^

A longer fluoroscopy period with high performance of the tube in the same irradiation geometry for cardiac procedures leads to high-dose skin spots and an increase in the possibility of transient erythema and even organ failure. Awareness of interventional cardiologists as regards the physical and anatomical conditions can help minimize the risk of skin spots by utilizing the best rotation in the tube so that there is more distribution in the patient’s body.^37^^, ^^38^

***Removal of the anti-scatter grid***

The role of the anti-scatter grid is to attenuate the scattered radiation and prevent it from entering the image receptor. For patients with a higher mass, an obligation is present for the removal of the scatter and preserving subject contrast in the image for enough image quality. Since scatter contributes to the brightness of the monitor image, primary radiation must be used instead of scatter. Primary radiation will increase the patient radiation dose 2 or more times.^39^

In pediatric cases, the removal of the grid could result in a dose decrease of up to 1/3 to 1/2, without showing any degradation in the quality and contrast of the image. Therefore, for children, grids should be used with caution in fluoroscopic examinations and the systems should have easy removal/introduction of the grid facility.^24^^, ^^40^

***Image magnification***

Fluoroscopes have 3 or more field of views (FOVs) on the display monitor, allowing the operator to select 1 of them. The biggest FOV is provided by the “normal” mode in that it irradiates in all the surface area of the image receptor. Other modes (e.g., mag 1, mag 2, etc.) utilize shorter X-ray beam areas at the receptor. Because smaller areas are expanded to fill the entire display monitor, the image of the anatomy is magnified for smaller FOVs.^41^ The sharpness of the image is also higher in magnified FOVs. The operator should be aware that when the FOV decreases, the radiation dose rate in fluoroscopy increases. It is dependent on the design of the machine. A bisecting dose 1/FOV or dose 1/FoV2 would increase the radiation dose rate by factors of 2 and 4, respectively.^24^^, ^^27^^, ^^39^

For the magnification of the image in fluoroscopy, 2 basic ways are employed: geometric and electric magnifications. In geometric magnification, the diverging X-ray is used to project a smaller area from the patient’s body to a larger region on the image intensifier.^42^^, ^^43^ When the distance from the source to the image receptor is constant, as the patient moves closer to the source, image magnification as well as skin dose will increase. Modern fluoroscopes have an option for image magnification within the image intensifier, electronically. Systems typically have electronic magnification modes, with each one having a unique dose level, and the dose increases with larger electronic magnifications.^41^ A rule of thumb is that the radiation dose to the patient increases by the square of the ratio of the image intensifier diameters. For example, if the entrance skin exposure is 100 units for a 23-cm FOV, the radiation entrance dose is augmented to 235 units, once the FOV is decreased to 15 cm (23/15) 2 and to 440 units for an FOV of 11 cm (23/11) 2.^27^^, ^^39^ Recently developed flat panel angiography, which has the advantage of pixel size enhancement, could perform large gain, flexible optical output with different types of optical cameras.^44^

***Digital subtraction angiography***

State-of-the-art interventional fluoroscopy sets have 2 properties: unlimited fluoroscopy time and the potential for performing digital subtraction angiography (DSA). DSA is used for diagnosis and documentation; it produces about twofold the radiation dose of the fluoroscopy-guided portion of the process. In these procedures, the total radiation dose can be substantially decreased either by storing fluoroscopy loops or extracting representative images from fluoroscopy loops instead of storing higher-quality DSA images in the recent generations of angiography systems.^26^^, ^^45^^, ^^46^

***Improving lead shielding***

Two effective strategies for the reduction of operator radiation exposure (down to 0.8% of the typical levels in catheterization laboratories) are radiation-attenuating interventional techniques and improved lead protection. For scatter radiation leakage, fitting a 1.0-mm lead top along the under-couch lead shield, and introducing a 1.0-mm lead flap below the lead glass sheet adjacent to the table could be effective. For cranial LAO angulation (LAO 60^°^/ 20^°^), inducing intensive scatter entrance surface air kerma (ESAK) to the operator, the lead flap is very effective in comparison with the under-couch top. With the co-installation of the aforementioned shields, we can reduce the mean scatter entrance skin air kerma to the operator position (S-ESAK-O) in this projection to a level of 0.7% of the primary scatter radiation. The angulation results in the mean S-ESAK-O reaching 2.6 to 15.8 Sv/h at the former leakage height.^47^ Three main types of shielding are equipment-mounted shields, personal protective devices, and architectural shielding. The last one is employed in the walls of the room. Moreover, additional shielding for operators, nurses, and anesthesia personnel is provided by other shields such as stationary and rolling ones.^20^

Previous reports have documented an average annual effective dose of 46.2 mSv for interventional cardiologists not wearing protective devices. By using a lead pinafore alone, the dose will reduce to 3.5 mSv annually, and by both a lead pinafore and a thyroid shield to 1.7 mSv.^5^

The most radiosensitive tissue in the body is the lens. A deterministic effect of radiation exposure (a threshold of 2 Gy) is cataract. The threshold for cataract development is reported by some investigations to be 0.5 Gy, which is less than the previous estimations. According to the International Commission on Radiological Protection reports, threshold values for detectable opacities of acute exposure and chronic exposure are 0.5 to 2.0 and 5 Sv, respectively.^48^

Ceiling-suspended lead shields could be replaced with leaded eye-protective glasses, which have protective side shields and show acceptable safety compared to other glasses. There are 4 personal protective shields: gloves, glasses, aprons, and thyroid shields. A 0.5-mm lead thickness apron or thyroid shield can reduce more than 95% of X-rays used during fluoroscopy. Leaded glasses can reduce from 35% to 95% of X-rays of typical X-ray energies. Ceiling-suspended shields can attenuate the operator dose by a factor of 3 to 20.^28^^, ^^49^^-^^52^

## Conclusion

Interventional cardiology is associated with some of the highest radiation doses in diagnostic radiology. We must assume that the practice of radiation protection with respect to both patients and operators needs improvement. The risk to patients and staff associated with these procedures can be kept to a minimum if operators maintain a diligent approach to radiation protection. If interventionists adopt the dose reduction techniques already available, particularly with reference to fluoroscopy, significant dose reduction can be achieved. Awareness of one’s own radiation dose is essential inasmuch as it provides enough motivation for incorporating changes in personal practice that result in reducing the radiation dose. The changes in the operator’s practice can be implemented very fast by a motivated operator at minimal or no cost and can have a substantial effect on the operator dose.

## Notes:

***This paper should be cited as:*** Mohammadi M, Danaee L, Alizadeh E. Reduction of Radiation Risk to Interventional Cardiologists and Patients during Angiography and Coronary Angioplasty. J Teh Univ Heart Ctr 2017;12(3):101-106.
